# Financial abilities in patients with Parkinson’s disease and mild cognitive impairment: unveiling cognitive and neurofunctional correlates of basic and advanced financial skills

**DOI:** 10.3389/fnagi.2026.1746491

**Published:** 2026-03-04

**Authors:** Laura Danesin, Giulia Pagnin, Lorenza Maistrello, Giorgia Baron, Arianna Menardi, Elena Piazzalunga, Alina Menichelli, Tatiana Cattaruzza, Leonardo Rigon, Konstantinos Koutsikos, Roberta Biundo, Giulio Ferrazzi, Carlo Semenza, Angelo Antonini, Paolo Manganotti, Antonino Vallesi, Francesca Burgio

**Affiliations:** 1 IRCCS San Camillo Hospital, Venezia, Italy; 2 Padua Neuroscience Center, University of Padua, Padua, Italy; 3 Defitech Chair of Clinical Neuroengineering, Neuro-X Institute (INX), cole Polytechnique Fédérale de Lausanne (EPFL), Geneva, Switzerland; 4 Defitech Chair of Clinical Neuroengineering, INX, EPFL Valais, Clinique Romande de Réadaptation, Sion, Switzerland; 5 Department of Neuroscience, University of Padua, Padua, Italy; 6 University School for Advanced Studies IUSS Pavia, Pavia, Italy; 7 Neuropsychological Service, Rehabilitation Unit - University Hospital and Health Services of Trieste, ASUGI, Trieste, Italy; 8 Neurology Unit, Hospital Care Department of Medicine, Azienda Sanitaria Universitaria Giuliano Isontina, Trieste, Italy; 9 Department of General Psychology (DPG), University of Padua, Padua, Italy; 10 Study Center for Neurodegeneration (CESNE), University of Padua, Padua, Italy; 11 Philips Healthcare, Milan, Italy; 12 Neurodegenerative Disease Unit, Department of Neuroscience, Padua Neuroscience Center (PNC), University of Padua, Padua, Italy; 13 Neurology Unit, Department of Medical, Surgical and Health Sciences, University of Trieste, Trieste, Italy

**Keywords:** cognitive functions, cognitive impairment, financial abilities, fMRI, functional connectivity, MRI, Parkinson’s disease

## Abstract

**Background:**

Parkinson’s disease (PD) entails widespread neurodegenerative changes extending beyond motor symptoms to cognitive and large-scale network alterations that compromise functional autonomy. Financial abilities (FAs) are complex, ecologically relevant skills crucial for independent living, yet their neurocognitive and neurofunctional substrates in PD remain largely unexplored. This study investigates the cognitive, structural, and neurofunctional correlates of basic and advanced FAs in PD with mild cognitive impairment (PD-MCI), using voxel-based morphometry to identify structural brain changes associated with FAs and resting-state network analyses to elucidate how brain connectivity supports preserved financial functioning.

**Methods:**

Thirty three individuals with PD-MCI completed a comprehensive neuropsychological assessment, including the Numerical Activities of Daily Living-Financial Short battery, to evaluate basic and advanced FAs. A subset of patients (*n* = 24) underwent acquisition of 3T structural and resting-state functional neuroimaging data. To identify cognitive and neural predictors of basic and advanced FAs, multiple regression models incorporating demographic covariates, cognitive and neuroimaging predictors were employed via stepwise Akaike Information Criterion and LASSO procedures.

**Results:**

Basic FAs were associated with general cognition and formal numerical competence (i.e., arithmetic knowledge), alongside negative functional correlations between somatomotor and subcortical networks. Advanced FAs were associated with different cognitive functions, such as executive ones, informal numerical competencies (i.e., use of numbers in everyday life), social cognition, language, and memory, and were linked to cerebellar network dynamics, specifically, increased anti-correlation with salience and limbic systems and enhanced synchronization with frontoparietal and subcortical circuits.

**Discussion:**

FAs in PD-MCI rely on a dynamic balance between network specialization and compensatory integration, reflecting adaptive reorganization of cortico-subcortical and cerebellar systems that may sustain complex cognitive functioning and functional independence.

## 1 Introduction

Parkinson’s disease (PD) is one of the most common neurodegenerative disorders (de Lau and Breteler, 2006), characterized by core motor difficulties such as bradykinesia, resting tremor, rigidity, and postural instability (Wu et al., 2012). In recent decades, the literature has increasingly focused on the non-motor symptoms of PD, including psychiatric concerns, sensory deficits, circadian rhythm disruptions, and cognitive impairment (De Rui et al., 2020), which can significantly affect patients’ quality of life and incur high healthcare costs (Silva et al., 2023). Notably, non-motor symptoms can occur before motor ones, with nearly 20% of patients showing mild cognitive impairment (MCI) at diagnosis (Aarsland et al., 2021). Of note, a recent meta-analysis of prospective population-based studies identified that individuals later diagnosed with PD showed baseline MMSE scores on average 0.3 points lower than healthy controls (Peña Arauzo et al., 2025). The primary cognitive deficits in PD patients involve attention, processing speed, working memory, set-shifting, and planning, along with difficulties in visuospatial skills, memory, language, or numerical competencies (Barone et al., 2011; Burgio et al., 2022b), ultimately disrupting the patients’ ability to independently perform daily tasks (Hiseman and Fackrell, 2017), such as taking medications or managing finances.

Notably, financial abilities (FAs) are fundamental to an individual’s independence and autonomy in everyday life (Marson et al., 2000), encompassing both basic practical tasks (e.g., counting currency) and advanced skills (e.g., managing household finances or identifying fraud attempts). Clinically, this distinction mirrors increasing functional complexity, with basic FAs reflecting simpler, routine financial tasks and advanced FAs requiring higher-order cognitive processes such as planning, judgment, and decision-making. However, even basic financial skills rely on preserved attentional, executive, and numeracy abilities, underscoring that financial functioning primarily reflects instrumental activities of daily living (IADL) rather than basic ADL (for a comprehensive overview, see also Marson, 2016). From a conceptual perspective, financial abilities are not a unitary construct, but rather encompass multiple, partially dissociable components spanning knowledge, skills, judgment, and decision-making processes. Influential capacity-based frameworks conceptualize financial abilities as part of decisional capacity, emphasizing an individual’s ability to understand financial information, appreciate its relevance to one’s personal situation, reason about available options, and communicate a choice (Appelbaum et al., 2016; Grisso and Appelbaum, 1998). Similarly, cognitive-functional models of financial capacity distinguish between foundational skills (e.g., basic numeracy, currency handling) and higher-order abilities that require complex integration of cognitive, emotional, and contextual information, such as financial judgment, planning, and fraud detection (Marson, 2016). More recently, integrative models have highlighted that real-world financial decision-making depends not only on classical cognitive domains but also on numerical competence, risk evaluation, and socio-cognitive processes, particularly in aging and neurodegenerative conditions (Callis et al., 2023).

In the context of Parkinson’s disease, previous studies have identified deficits in basic areas of FAs in patients both with and without cognitive impairment (Martin et al., 2013). In contrast, advanced FAs seem to be relatively preserved even in patients with PD and dementia (Martin et al., 2013). Interestingly, no significant correlation has been identified between FAs and cognitive performance in patients with PD (Pirogovsky et al., 2013, 2014). However, prior studies have predominantly focused on classical cognitive domains (i.e., attention, memory, executive functions, language, and visuospatial abilities) and have not investigated other functions that may be implicated in FAs, such as numerical competence or social cognition. In fact, deficits in these areas have been reported in the literature (Burgio et al., 2022b; Palmeri et al., 2017), but to date, they have not been directly explored in relation to FAs.

Moreover, the functional neural underpinnings of FAs remain scarcely studied in this population. Resting-state functional MRI (fMRI) is widely used to characterize intrinsic large-scale functional connectivity supporting cognitive functions and enables the investigation of network-level organization independent of task performance and motor confounds, which are relevant in PD-MCI. Notably, several studies have observed functional reorganization in large-scale resting-state networks in patients with PD (Tessitore et al., 2019), particularly in the attentive, executive, and default mode (DMN) networks (Menon, 2011; Sang et al., 2015). Decreased connectivity within the DMN has been identified as a core feature in patients with PD-MCI or dementia (Díez-Cirarda et al., 2018; Wolters et al., 2019). Similarly, disrupted connectivity between the attentive [i.e., dorsal attention network (DAN); salience ventral attention network (SVAN)] and executive [control network (CON)] networks, as well as decreased connectivity within the visual network (VIS), appears to affect cognitive performance (Baggio et al., 2015; Hou et al., 2021; Putcha et al., 2015).

In addition to functional network alterations, structural connectivity in PD-MCI has been widely studied using voxel-based morphometry (VBM), which is a powerful whole-brain approach for detecting structural brain alterations associated with cognitive impairment. Structural MRI studies have revealed consistent gray matter (GM) atrophy in regions linked to executive memory and function, including the prefrontal cortex, insular, and striatal structures (Lee et al., 2014; Wang et al., 2025). By employing VBM, it is possible to identify spatially distributed patterns of atrophy that are linked to cognitive decline in PD-MCI, thereby elucidating the neuroanatomical basis of cognitive impairment and disease progression (Burgio et al., 2022b; Summerfield et al., 2005).

However, to the best of our knowledge, no previous study has directly examined the relationship between the structural changes and functional network alterations in financial management in this population. Therefore, the primary aim of the present exploratory study is to investigate the neurocognitive correlates and predictors of FAs in patients with PD-MCI, shedding light on the mechanisms underlying both basic and advanced FAs. In particular, as individuals with PD-MCI may exhibit difficulties in numerical competencies (Burgio et al., 2022b), we aim to expand previous findings on the cognitive predictors of FAs, disentangling the association between numerical competencies and the basic and advanced components of FAs in this population. Moreover, we aim to explore whether different structural and functional network features may be involved in basic and advanced FAs, to generate the basis for additional hypotheses to be tested in future studies. Consistent with FAs models (Callis et al., 2023; Marson, 2016), we operationalized financial abilities along a continuum from basic to advanced tasks. We hypothesized that basic FAs would correlate primarily with classical cognitive domains and numerical competence, whereas advanced FAs would show stronger associations with integrative executive and decision-making processes. Furthermore, given evidence of both functional network disruptions and structural atrophy in PD-MCI, we hypothesized that distinct neural correlates underlie basic vs. advanced FAs, with global and network-level alterations more strongly associated with advanced FAs.

## 2 Materials and methods

The present study is part of a clinical trial registered on Clinicaltrials.gov (NCT05826548).

### 2.1 Participants

Thirty three patients with PD-MCI (10 females, 23 males) were recruited from patients admitted to IRCCS San Camillo Hospital or referred for clinical screening due to suspected cognitive impairment from September 2021 to February 2025. Participants had a mean age of 72 years (SD = 9) and a mean education of 11 years (SD = 4). Inclusion criteria were: (i) age at onset 40–85 years old; (ii) diagnosis of PD-MCI (Litvan et al., 2012); (iii) absence of psychiatric illnesses and/or comorbidity with other neurological pathologies; (iv) ability to provide informed consent.

Patients underwent cognitive and clinical assessments, as well as neuroimaging data acquisition. All evaluations were performed by experienced and trained neuropsychologists. Evaluations were conducted preferably during the morning, during the ON period of patients’ usual dopaminergic therapy (details on patients’ pharmacotherapies are reported in Supplementary material).

All participants in the study voluntarily participated and provided informed consent, in accordance with the principles outlined in the Declaration of Helsinki. The study was approved by the Ethics Committee of Venice and IRCCS San Camillo Hospital (Venice, Italy), reference number 1081/IRCCS San Camillo.

### 2.2 Cognitive assessment

Each patient completed a comprehensive neuropsychological evaluation, which included measures of attention, executive functions, memory, visuospatial abilities, calculation, language, and social cognition. In particular, the following tests were administered: (i) Mini-Mental State Examination (MMSE) (Magni et al., 1996) and Montreal Cognitive Assessment (MoCA) (Santangelo et al., 2015) for general cognitive functioning; (ii) Trail Making Test (TMT) (Giovagnoli et al., 1996) for attention; (iii) Stroop test (Caffarra et al., 2002a), Clock Drawing Test (CDT) (Mondini et al., 2003), and Phonological fluencies (i.e., naming of F-words, A-words and S-words) (Carlesimo et al., 1996) for executive functions; (iv) Rey Auditory Verbal Learning Test (RAVLT) (Carlesimo et al., 1996), Prose memory (Spinnler, 1987), and recall of the Rey-Osterrieth Complex Figure (ROCF-recall) (Caffarra et al., 2002b) for learning and memory domain; (v) copy of the Rey-Osterrieth Complex Figure (ROCF-copy) (Caffarra et al., 2002b) for the visuospatial and visuoconstructive domain; (vi) Semantic fluencies (i.e., naming of fruits, animals and car brands) (Novelli et al., 1986) for the language domain; (vii) the Numerical Activities of Daily Living Short version (NADL) (Burgio et al., 2022a) for informal (i.e., use of numbers in everyday situations) and formal numerical competencies (i.e., scholastic arithmetical knowledge); (viii) the Story-Based Empathy Task (SET) (Dodich et al., 2015) for social cognition.

### 2.3 Evaluation of financial abilities

Patients’ financial abilities were assessed using the Numerical Activities of Daily Living-Financial Short version (NADL-F) (Toffano et al., 2021). The NADL-F was validated in a heterogeneous sample of neurological patients and is designed to assess seven components of financial abilities, ranging from daily tasks (e.g., counting currencies) to advanced skills (e.g., financial judgments) related to higher-order cognitive functioning. Specifically, the seven subscales are the following:

1. Counting Currencies (maximum score = 3) evaluates whether the participant is familiar with the Euro currency and is able to perform simple mental calculations, analogous to those involved in simple cash transactions, but in a simplified setting (e.g., the patient is given an amount of coins and bills and is asked to count them out loud).
2. Reading Abilities (maximum score = 3) assesses whether the participant is able to deal with written information about money in everyday life situations (e.g., the patient is asked to check the cost of some items on a supermarket receipt).
3. Item Purchase (maximum score = 4) assesses the participant’s ability to perform operations (e.g., calculations, considering relevant information) necessary for making cash transactions during real-life shopping (e.g., the patient is given an amount of coins and bills and is asked to give the examiner the amount needed to pay for some fruits).
4. Percentages (maximum score = 3) assesses whether the participant can perform mental calculations with percentages in real-life contexts (e.g., the patient is asked to calculate the discounted price of some clothes).
5. Bill Payments (maximum score = 2) evaluates knowledge regarding managing bills (e.g., the patient is asked to organize in chronological order some bills on the basis of the due payment dates).
6. Financial Concepts (maximum score = 6) assesses the participant’s knowledge of financial concepts that are relevant in the Italian cultural context (e.g., the patient is asked to provide a description of International Bank Account Number (IBAN), or Indicatore della Situazione Economica Equivalente (ISEE), a tool used in Italy to assess a family’s economic situation for accessing subsidized social benefits).
7. Financial Judgments (maximum score = 2) assesses whether the participant can make informed financial judgments and identify fraudulent behaviors (e.g., the examiner presents the patient with a short story and asks them to assess whether the character is being defrauded).

### 2.4 Neuroimaging

#### 2.4.1 Data acquisition

Structural MRI was acquired to identify structural brain changes associated with Fas. Resting-state fMRI was performed to characterize intrinsic large-scale functional connectivity supporting financial abilities.

Structural and functional MRI data were collected for a subset of participants (*n* = 24) using a 3T Philips Ingenia scanner (Philips Medical Systems, Best, The Netherlands) with a 32-channel receiver head coil. Nine patients were excluded due to clinical and/or safety concerns (claustrophobia, presence of a pacemaker, metallic prosthesis, etc.).

The T1-weighted [(3-dimensional Magnetization Prepared T1 weighted Rapid Gradient Echo (MP-RAGE)] anatomical images were acquired at 0.8 mm^3^ spatial resolution, repetition time (TR) = 10 ms, echo time (TE) = 4.6 ms, inversion time (TI) = 950 ms, flip angle (FA) = 8 degrees. Resting-state functional MRI data were acquired at 1.96 × 1.96 × 2.4 mm^3^ spatial resolution, TR = 2.1 s, TE = 30 ms, number of slices = 60, number of volumes = 421, flip angle = 90 degrees, multiband factor = 3, SENSE factor = 1.2.

#### 2.4.2 Preprocessing and analysis

Structural preprocessing included bias field correction (ANTs), brain extraction (ANTs), and tissue segmentation [Computational Anatomy Toolbox; segmentation into GM, white matter (WM), and cerebrospinal fluid (CSF)].

Functional preprocessing comprised slice timing correction, motion correction (MCFLIRT of FSL) (Jenkinson et al., 2012), distortion correction (TOPUP of FSL), denoising steps including confound regression (WM and CSF, motion parameters, and outlier scans identified with ART) through CONN toolbox (Whitfield-Gabrieli and Nieto-Castanon, 2012), high-pass filter (0.01 Hz), ICA-based artifact removal using ICA-FIX (Griffanti et al., 2014; Salimi-Khorshidi et al., 2014), as well as low-pass filter (0.1 Hz). Participants with a framewise displacement higher than 0.5 mm were excluded.

Voxel-based morphometry (VBM) indices were derived from T1-weighted anatomical images using the Computational Anatomy Toolbox (CAT12 toolbox) (Gaser et al., 2024) to quantify variations in brain tissue composition (specifically GM, WM, and CSF).

**FIGURE 1:**
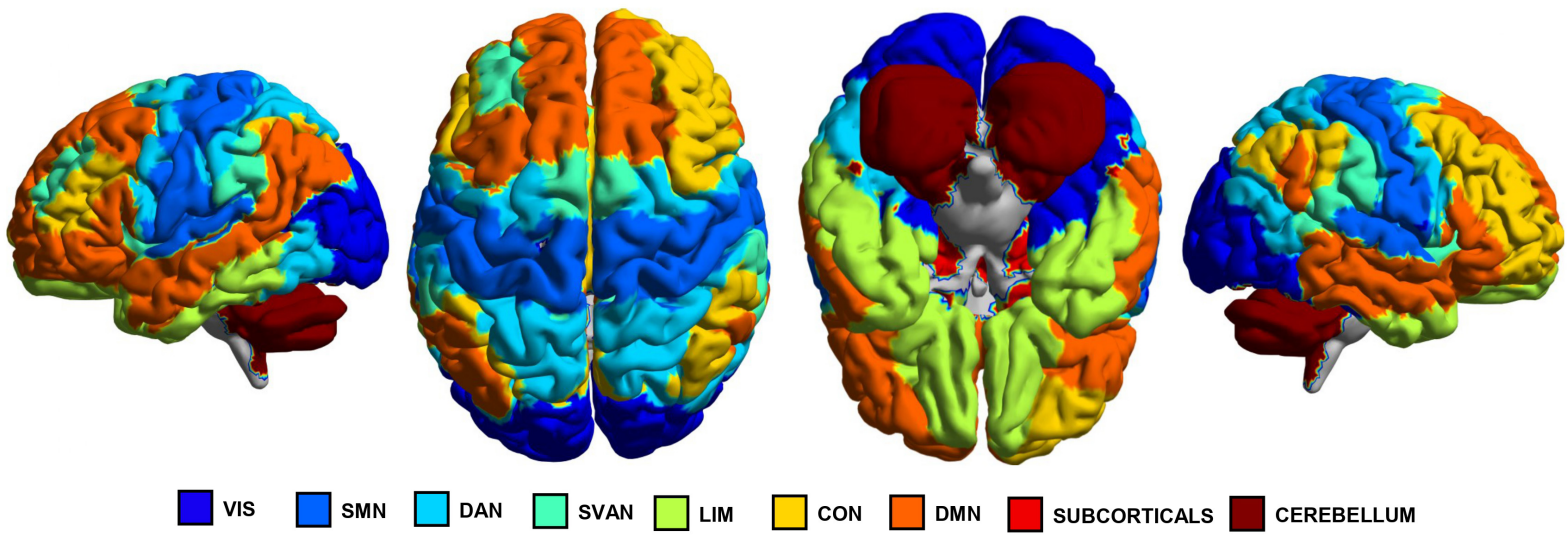
Graphical representations of the brain regions involved in each functional network, as identified by the Schaefer’s and AAL3’s atlases, highlighted in different colors. SMN, somatomotor network; VIS, visual network; DAN, dorsal attention network; SVAN, salience ventral attention network; LIM, limbic network; CON, Control network; DMN, default mode network.

The cerebral cortex of each patient was then divided into 100 regions of interest (ROIs) based on the functional atlas of interest (Schaefer et al., 2018), which includes seven functional networks: VIS, Somatomotor (SMN), DAN, SVAN, Limbic (LIM), CON, and DMN. Additionally, 10 subcortical regions were extracted from the AAL3 atlas (Rolls et al., 2020), together with the cerebellum (see Figure 1 for a graphical representation of the different brain regions involved in each network). Time series were extracted for each brain region in the parcellation by averaging the preprocessed blood oxygen level-dependent (BOLD) fMRI signal within each node at each time point, and then correlated using Pearson’s linear correlation to obtain a 112-by-112 functional connectivity (FC) matrix containing the correlation index between each pair of regions. The z-Fisher transformation was applied to the obtained FC matrices, and the values were scaled to the range [0, 1].

Graph metrics, including average node strength and global efficiency, were selected to capture complementary aspects of network organization (Bullmore and Sporns, 2009; Luo et al., 2015; Tinaz et al., 2017). Those metrics were computed using the Brain Connectivity Toolbox (BCT) (Rubinov et al., 2009). Node strength is defined as the sum of weights of all edges connected to a given node, then averaged across networks. It reflects the overall level of connectivity or influence of that region within the network. Then, global efficiency, defined as the inverse of the average path length between all pairs of nodes, was calculated on the scaled FC matrices, with higher values indicating more integrated and efficient communication across the network. The average path length refers to the mean of the shortest paths connecting every pair of nodes in the network, providing a measure of how easily information can travel across the network. Shorter average path lengths indicate that nodes can communicate more efficiently, reflecting a more integrated network organization.

In addition to global metrics, within- and between-network connectivity measures were computed (Boon et al., 2020). Within-network connectivity refers to the average FC across nodes belonging to the same functional network (e.g., DMN, CON). It reflects the cohesion and integrity of a given network. Between-network connectivity, on the other hand, measures the average connectivity between regions across different networks and is typically used to assess the degree of functional segregation or integration between brain systems.

### 2.5 Statistical analysis

All statistical analyses were performed using the open-source software RStudio (version 4.5.1) (R Core Team, 2020). Statistical significance was set at *p* < 0.05. Missing data were present for some variables; however, for all variables the proportion of missing values was below 25%. Accordingly, missing data were imputed using multivariate imputation by chained equations (MICE), which preserves the underlying distribution of the data (Nich and Carroll, 2002).

The main characteristics of the sample were analyzed using descriptive statistics, i.e., mean and standard deviation (SD) for quantitative variables, and absolute frequencies (*n*) and percentages (%) for qualitative variables.

Participants’ raw scores on the neuropsychological tests were first corrected for demographic characteristics (age, education, and/or gender) based on the normative rules for each test, and were then converted into *z*-scores based on a sample of age- and education-matched healthy controls. Healthy controls’ data were drawn from the NADL-F and NADL validation datasets (Arcara et al., 2019; Semenza et al., 2014) (see Supplementary material for additional information). The patients’ *z*-scores were then averaged to form 7 composite variables representing each cognitive domain (general cognitive functioning, attention, executive functions, memory, language, visuospatial abilities, social cognition). Similarly, *z*-scores were computed for the informal and formal components of the NADL test. NADL-F subtests were summed to create basic and advanced scores of FAs and then *z*-scored based on the performance of healthy controls. Specifically, the basic FAs domain comprised the “Counting currency,” “Reading abilities,” “Item purchase,” and “Percentages” subtests, while the advanced FAs domain comprised the “Financial concepts,” “Bill payments,” and “Financial Judgments” subtests. An impaired performance was defined as a *z*-score equal to or below −1.5.

Initial data analysis was conducted using non-parametric partial correlations to investigate the interdependencies among the variables, while accounting for the potential influence of age, sex, and years of education on test outcomes. This approach was chosen due to the small sample size (*N* = 33) and the exploratory nature of the study, allowing us to detect potential associations without relying on parametric assumptions. The resulting correlation coefficients (rho) provide a robust estimate of relationships independent of demographic influences. Cognitive composite scores were correlated with the continuous scores on the NADL informal and formal tests, as well as with NADL-F basic and advanced scores. Similarly, cognitive, NADL, and NADL-F scores were correlated with VBM and functional metrics. The interpretation of correlation coefficients followed the classification proposed by Schober et al. (Schober et al., 2018): rho < 0.10 = negligible; 0.10 < rho < 0.39 = weak; 0.40 < rho < 0.69 = moderate; 0.70 < rho < 0.89 = strong; rho > 0.90 = very strong.

Then, to identify cognitive and neural predictors associated with difficulties in basic and advanced financial skills, multiple regression models were first constructed using cognitive variables (numerical abilities, general cognitive functioning, attention, executive functions, memory, language, visuospatial abilities, and social cognition) as predictors, and financial abilities as continuous dependent variables. Age, gender, and level of education were included as covariates. Multicollinearity among predictors was assessed using the Variance Inflation Factor (VIF), with VIF > 5 indicating high multicollinearity, which can lead to unstable regression coefficients (Hair et al., 2019). To select the optimal set of predictors, stepwise procedures based on the Akaike Information Criterion (AIC) and penalized regression models (LASSO) were employed. The predictive accuracy of the models was evaluated using 10-fold cross-validation (James et al., 2021).

Lastly, limited to the subgroup of patients with available neuroimaging data, extended models were developed by including brain variables, such as measures of structural and FC. The extended models were compared to the previous ones to assess the incremental contribution of neural variables in explaining financial performance.

## 3 Results

### 3.1 Clinical characteristics of the sample

At the cognitive evaluation, participants showed slight impairments on the two screening tests, with average scores of 27.51 (SD = 2.02) and 23.06 (SD = 5.46) for the MMSE and MoCA, respectively. From a qualitative point of view, some patients exhibited impairments in general cognitive functioning (30.3%), attention (24.2%), executive functions (24.2%), memory (24.2%), language (24.2%), visuospatial abilities (15.2%), and social cognition (18.2%). In the NADL test, 12.1% of patients had difficulties with formal numerical competencies, while only 1 patient (3%) had difficulties with the informal ones. Lastly, at the NADL-F test, 18.2% of participants were impaired in basic FAs, while 12.1% were impaired in advanced FAs. Table 1 reports the main demographic and clinical characteristics of the sample. Notably, no significant differences were observed between patients with neuroimaging data and the entire sample.

**TABLE 1:** Sociodemographic characteristics and cognitive profile of the patients enrolled in the study.

Characteristics	Whole sample (*N* = 33)	Neuroimaging subsample (*N* = 24)	U/X^2^ (*p*-value)
Age, years	72 (± 9)/71 [13]	74 (± 8)/74 [13]	346 (0.422)
Education, years	11 (± 4)/11 [5]	10 (± 4)/11 [8]	349 (0.446)
Gender, *n*	10 F, 23 M	8 F, 16 M	0.059 (0.808)
Disease duration, years	8.67 (± 5.9)/7.79 [10.00]	8.12 (± 5.45)/8.25 [9.29]	208 (1.000)
Leovodopa equivalent daily dose, mg	827.88 (± 500.21)/650 [517.32]	712.75 (± 445.65)/567 [267.36]	284 (0.307)
MMSE, score	27.5 (± 2.02)/27.86 [2.96]	27.6 (± 2.17)/28.15 [2.96]	380 (0.801)
MoCA, score	23.1 (± 5.46)/24.58 [7]	23.0 (± 6.05)/25.16 [7.71]	389 (0.910)
Global cognition, *z*-score	−0.68 (± 1.54)/-0.68 [1.37]	−0.70 (± 1.06)/-0.63 [1.61]	392 (0.955)
Attention, *z*-score	0.87 (± 0.42)/0.80 [0.13]	−0.67 (± 1.06)/0.83 [0.15]	367 (0.645)
Executive function, *z*-score	0.07 (± 0.77)/-0.04 [0.64]	−0.85 (± 1.32)/0.01 [0.71]	389 (0.916)
Memory, *z*-score	−0.81 (± 0.96)/-0.83 [1.43]	−0.89 (± 0.85)/-1.03 [1.63]	389 (0.910)
Language, *z*-score	−0.49 (± 1.44)/-0.55 [1.60]	−0.62 (± 1.53)/-0.84 [1.11]	392 (0.948)
Visuospatial abilities, *z*-score	−0.18 (± 1.19)/0.27 [0.70]	−0.59 (± 1.43)/0.29 [0.43]	385 (0.865)
Social cognition, *z*-score	−0.77 (± 1.43)/-0.50 [1.76]	−0.52 (± 1.36)/-0.69 [2.61]	145 (0.867)
Informal numerical competencies, *z*-score	0.18 (± 0.96)/0.31 [1.19]	0.51 (± 0.94)/0.20 [1.00]	134 (0.592)
Formal numerical competencies, *z*-score	−0.22 (± 0.84)/-0.26 [1.03]	−0.35 (± 0.94)/0.08 [1.03]	142 (0.802)
Basic FAs, *z*-score	−0.34 (± 1.95)/0.13 [1.42]	−0.49 (± 1.54)/0.07 [1.56]	391 (0.942)
Advanced FAs, *z*-score	0.16 (± 1.26)/0.24 [1.40]	0.13 (± 1.32)/0.07 [1.29]	342 (0.382)

Means and (±) standard deviations/median and interquartile range, or number of patients, are reported separately for the whole sample and for patients with neuroimaging data. Results of the Mann-Whitney U test or the Chi-square test (χ^2^) are reported for continuous and categorical variables, respectively, to assess whether the neuroimaging subsample differed statistically from the full sample in cognitive and clinical characteristics. MMSE, Mini-mental State Examination; MoCA, Montreal Cognitive Assessment; FAs, Financial Abilities.

### 3.2 Cognitive correlates of FAs

**FIGURE 2:**
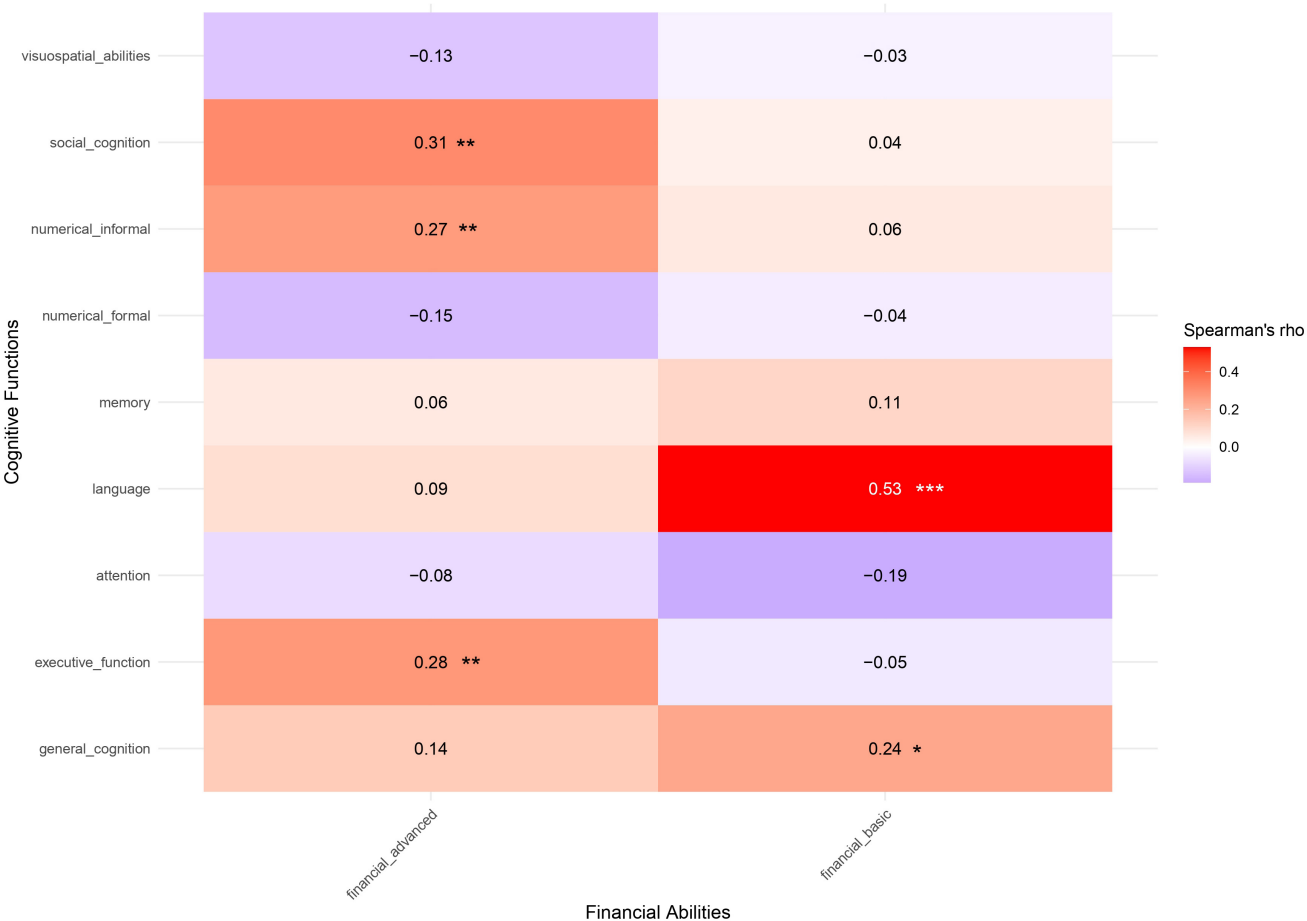
Spearman’s correlation coefficients between FAs components and cognitive functions. Positive correlations are depicted in red, negative ones in blue. Significant correlations are highlighted with an asterisk (***p < 0.001; **p < 0.01; *p < 0.05).

Figure 2 reports the correlations between FAs and cognitive measures. Basic FAs were moderately correlated with language (rho = 0.53, *p* < 0.001), while showing lower correlations with general cognitive functioning (rho = 0.24, *p* = 0.016). Advanced FAs showed low associations with cognition, namely executive functions (rho = 0.28, *p* = 0.006) and social cognition (rho = 0.31, *p* = 0.002).

Conversely, formal numerical competence was moderately correlated with general cognitive functioning (rho = 0.53, *p* < 0.001) and visuospatial abilities (rho = 0.56, *p* < 0.001), and showed low correlations with executive functions (rho = 0.20, *p* < 0.046) and memory (rho = 0.27, *p* = 0.008). On the other hand, informal numerical competence was correlated with general cognitive functioning (rho = 0.31, *p* = 0.002), language (rho = 0.32, *p* = 0.001), memory (rho = 0.29, *p* = 0.004), and social cognition (rho = 0.35, *p* < 0.001).

A significant correlation was observed between advanced FAs and informal numerical competencies (rho = 0.27, *p* = 0.008), while no significant correlation emerged between basic FAs and formal or informal numerical competencies (*p* > 0.050).

### 3.3 Neural correlates of FAs

**FIGURE 3:**
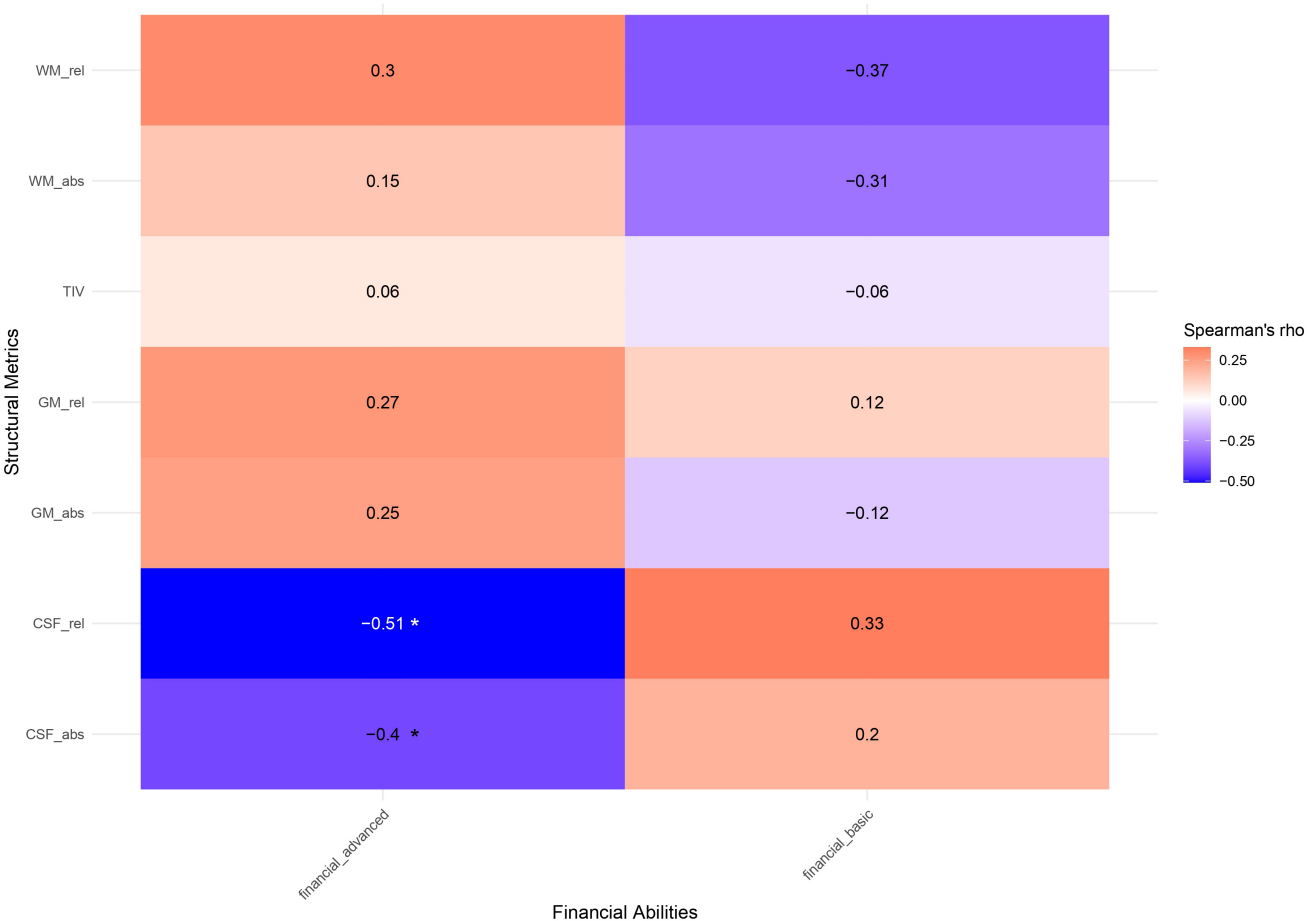
Spearman’s correlation coefficients between FAs components and structural neuroimaging metrics. Positive correlations are depicted in red, negative ones in blue. Significant correlations are highlighted with an asterisk (*p < 0.05). WM, white matter; GM, gray matter; CSF, cerebrospinal fluid; TIV, total intracranial volume; rel, relative volume; abs, absolute volume.

**FIGURE 4:**
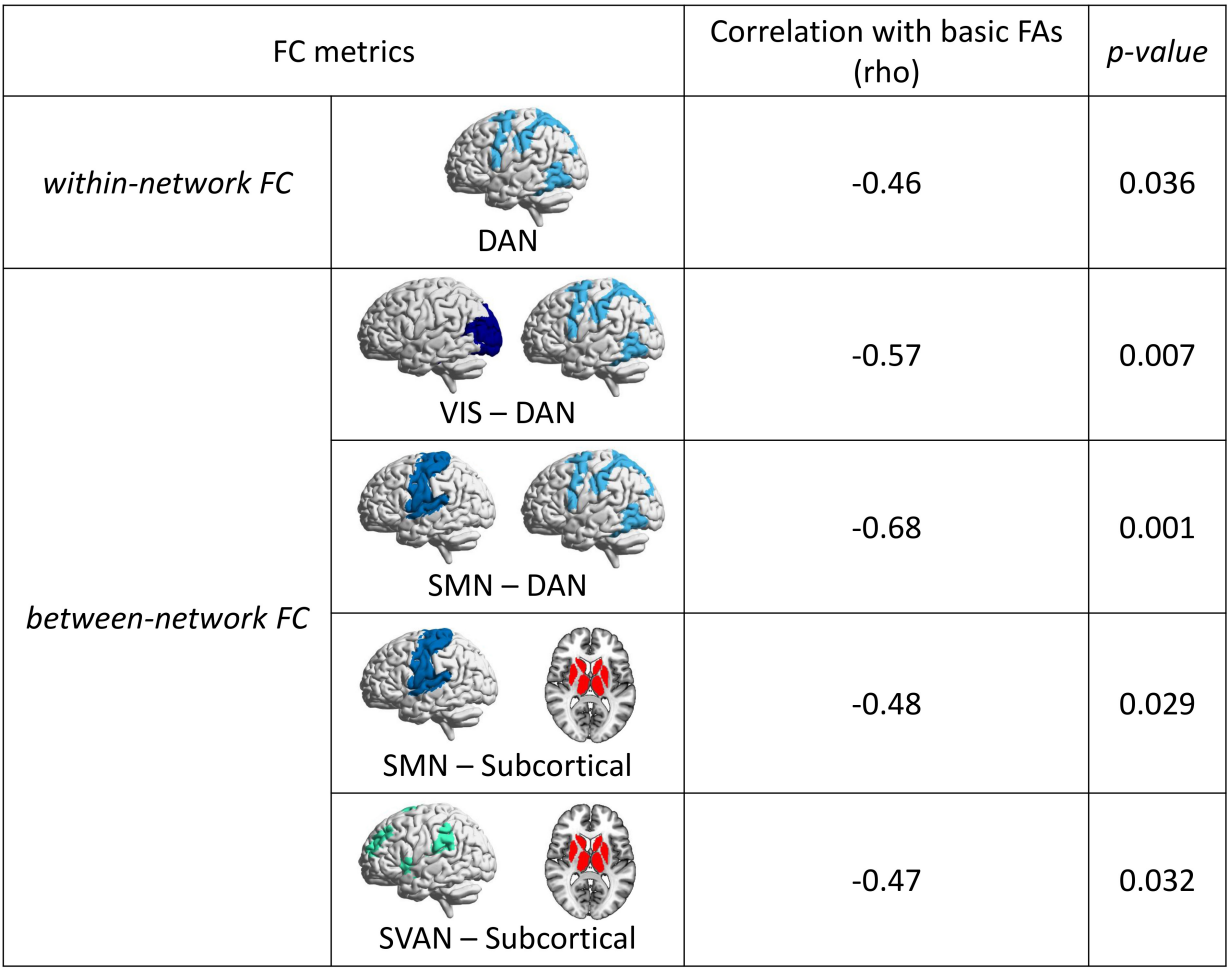
Significant correlations between basic financial abilities (FAs) and measures of functional connectivity (FC) for large-scale brain networks. Spearman’s rho values and *p*-values are reported. Notably, no significant correlation was observed between FC measures and advanced FAs. DAN, dorsal attention network; SMN, somatosensory network; SVAN, salience ventral attention network; VIS, visual network.

**FIGURE 5:**
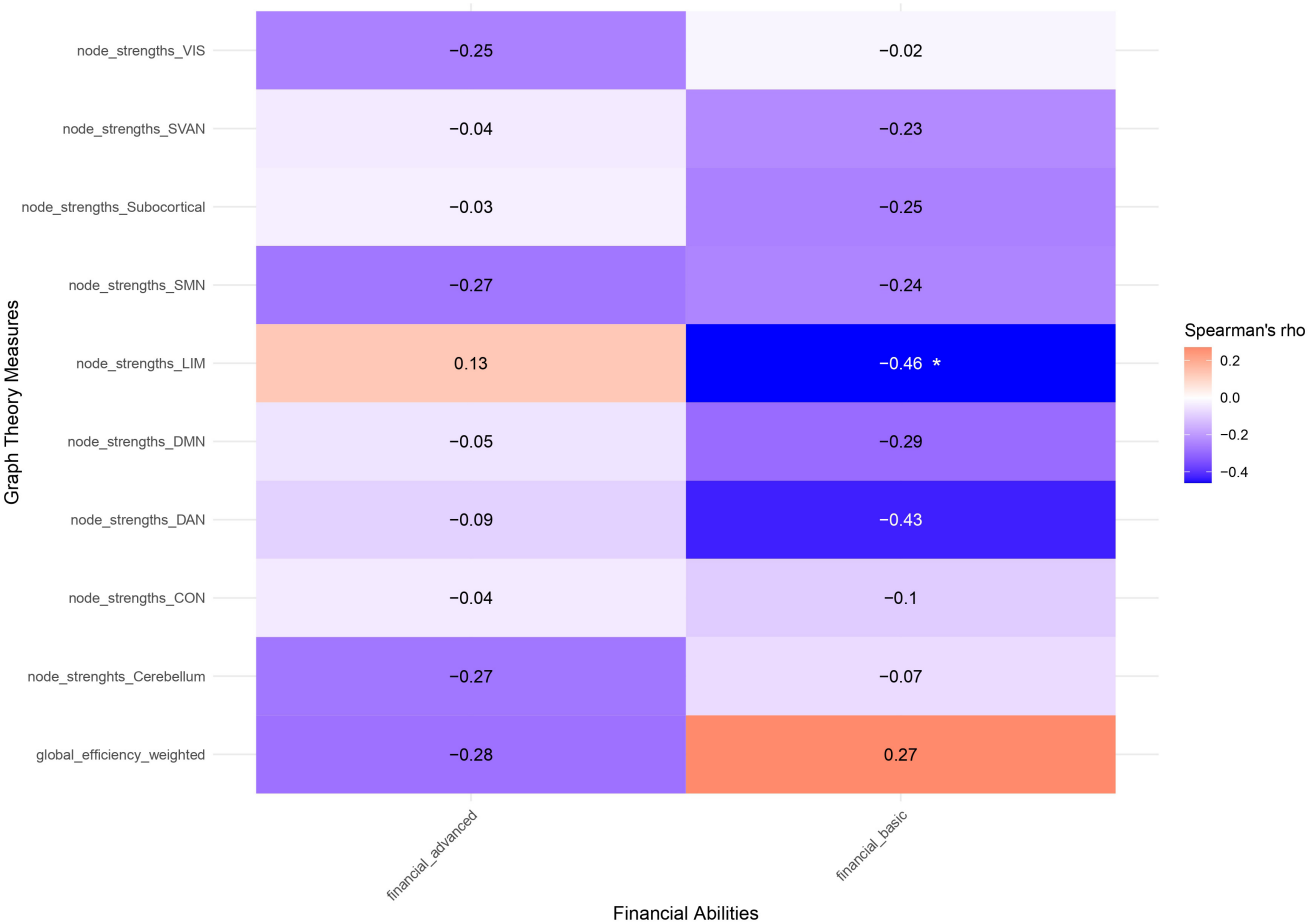
Spearman’s correlation coefficients between FAs components and graph theory metrics. Positive correlations are depicted in red, negative ones in blue. Significant correlations are highlighted with an asterisk (*p < 0.05). SMN, somatomotor network; VIS, visual network; DAN, dorsal attention network; SVAN, salience ventral attention network; LIM, limbic network; CON, Control network; DMN, default mode network.

Figures 3–5 report the correlations between FAs and neuroimaging measures. Regarding neuroanatomical metrics (Figure 3), advanced FAs were negatively correlated with CSF volume (rho = -0.51, *p* = 0.017), whereas no significant correlations emerged between the VBM metrics and basic FAs, formal or informal numerical competencies.

When considering the association between numerical competencies and functional metrics, informal numerical competence showed moderate negative correlations with within-network connectivity in the CON network (rho = -0.54, *p* = 0.012) and with the LIM-CON between-network connectivity (rho = -0.47, *p* = 0.033). Formal numerical competence was negatively correlated with within-network connectivity in the LIM (rho = -0.47, *p* = 0.030) and DMN networks (rho = -0.52, *p* = 0.015).

Basic FAs exhibited moderate negative correlations with within-network connectivity in the DAN (rho = -0.46, *p* = 0.036), and with the VIS-DAN (rho = -0.57, *p* = 0.007), SMN-DAN (rho = -0.68, *p* = 0.001), SMN-Subcortical (rho = -0.48, *p* = 0.029), SVAN-Subcortical between-network connectivity (rho = -0.47, *p* = 0.032). Significant correlations between basic FAs and within- and between-network connectivity of large-scale brain networks are presented in Figure 4. Moreover, a significant negative correlation was observed between basic FAs and node strength in the LIM (rho = -0.46, *p* = 0.037) (Figure 5).

Lastly, advanced FAs showed no significant correlations with functional metrics (see Supplementary Figures S1, S2 for a complete overview of the correlation coefficients between FC metrics and basic/advanced FAs).

### 3.4 Cognitive predictors of FAs

All cognitive and demographic variables were included in the preliminary models. Variance Inflation Factor (VIF) values remained below the critical threshold of 5 for both the basic financial abilities model (maximum VIF = 3.12) and the advanced financial abilities model (maximum VIF = 3.12), indicating negligible multicollinearity risk.

In the multiple linear regression model with LASSO regularization (Table 2), variance in basic FAs performance was moderately explained by cognitive and demographic predictors (*R*^2^ = 0.65; adj. *R*^2^ = 0.57). Among the predictors, the following factors emerged as significant: general cognition (β = 0.66, *p* = 0.002), formal numerical competence (β = -0.76, *p* = 0.021), and education (β = 0.23, *p* < 0.001). The model’s predictive accuracy yielded a root mean squared error (RMSE) of 1.77 (SD = 1.23) for the optimal penalty parameter (λ = 0.132).

**TABLE 2:** Results of the multiple linear regression analyses with LASSO regularization predicting financial abilities (FAs) using only cognitive variables.

Outcome	Predictor	Estimate	SE	*t*-value	*p*-value	
(a) Basic FAs	General cognition	0.66	0.19	3.48	0.002	**
	Social cognition	−0.36	0.18	−1.98	0.058	.
	Language	0.31	0.18	1.76	0.090	.
	Formal numerical competence	−0.76	0.31	−2.45	0.021	*
	Gender (male)	−0.78	0.54	−1.45	0.158	
	Education	0.23	0.06	3.85	0.001	***
(b) Advanced FAs	Executive function	0.30	0.18	1.65	0.072	.
	Age	−0.05	0.02	−2.53	0.012	*
	Education	0.12	0.04	2.70	0.007	**

For each model, the dependent variable (outcome), predictor variables, coefficient estimates (Estimate), standard errors (SE), *t*-values, and *p*-values are reported. Significance codes: “***” *p* < 0.001; “**” *p* < 0.01; “*” *p* < 0.05; “⋅” *p* < 0.1; “ ” *p* ≥ 0.1.

For the model concerning advanced FAs (Table 2), multiple linear regression analyses yielded an *R*^2^ of 0.49 (adj. *R*^2^ = 0.44). The most relevant predictors, identified via LASSO, were executive function (β = 0.30; *p* = 0.072), age (β = -0.05; *p* = 0.012), and education (β = 0.12; *p* = 0.007). The RMSE for the optimal penalty parameter (λ = 0.18) was 1.07 (SD = 0.16).

### 3.5 Neurofunctional predictors of FAs

In the subsample of patients with available neuroimaging data (*n* = 24), including FC measures significantly improved model fit for both basic (*R*^2^ = 0.84; adj. *R*^2^ = 0.81; Δ adj. *R*^2^ ≈ 0.24) and advanced FAs (*R*^2^ = 0.89; adj. *R*^2^ = 0.77; Δ adj. *R*^2^ ≈ 0.34). These results suggest that brain connectivity variables offer incremental explanatory value beyond cognitive and demographic predictors.

In multiple linear regression models that also included neuroimaging variables (Table 3), LASSO penalized regression (optimal λ = 0.43; RMSE = 1.87; SD = 2.25) revealed the following variables to be significant for basic FAs: language (β = 0.9; *p* < 0.001), SMN within-network connectivity (β = -9.33; *p* < 0.001), between-network connectivity in SMN-subcortical (β = -7.64; *p* < 0.001).

**TABLE 3:** Results of the multiple linear regression analyses with LASSO regularization for models predicting financial abilities (FAs) including cognitive and neuroimaging outcomes.

Outcome	Predictor	Estimate	SE	*t*-value	*p*-value	
(a) Basic FAs	Language	0.9	0.7	4.73	< 0.001	***
	SMN wn-FC	−9.33	1.22	−7.65	< 0.001	***
	SMN-subcorticals bn-FC	−7.64	1.76	−4.35	< 0.001	*
(b) Advanced FAs	Language	0.94	0.23	4.04	0.002	**
	Memory	−1.01	0.29	−3.55	0.005	**
	Formal numerical competence	0.2	0.21	0.94	0.37
	Age	0.09	0.03	2.88	0.016	*
	WM absolute	0.02	0.01	3.76	0.004	**
	GM relative	31.02	10.84	2.86	0.017	*
	WM relative	41.50	10.89	3.81	0.003	**
	SMN wn-FC	−4.93	1.03	−4.8	0.001	***
	SVAN-Cer bn-FC	−15.31	2.78	−5.5	< 0.001	***
	LIM-Cer bn-FC	−1.97	0.8	−2.47	0.033	*
	CON-Cer bn-FC	4.27	1.29	3.31	0.008	**
	Subcorticals-Cer bn-FC	6.82	1.76	3.88	0.003	**
	SMN node strength	0.14	0.03	4.42	0.001	**

For each model, the dependent variable (outcome), predictor variables, coefficient estimates (Estimate), standard errors (SE), *t*-values, and *p*-values are reported. Significance codes: “***” *p* < 0.001; “**” *p* < 0.01; “*” *p* < 0.05; “⋅” *p* < 0.1; “ ” *p* ≥ 0.1. SMN, somatomotor network; VIS, visual network; DAN, dorsal attention network; LIM, limbic network; CON, Control network; wn-FC, within-network functional connectivity; bn-FC, between-network functional connectivity; WM, white matter; GM, gray matter; Cer, cerebellum.

For advanced FAs model (Table 3), the most relevant predictors for LASSO penalized regression were: language (β = 0.93, *p* = 0.002), memory (β = -1.01, *p* = 0.005), age (β = 0.09, *p* = 0.016), WM absolute (β = 0.02, *p* = 0.004), GM relative (β = 31.02, *p* = 0.017), WM relative (β = 41.50, *p* = 0.003), SMN within-network connectivity (β = -4.93, *p* = 0.001), SVAN-Cerebellum between-network connectivity (β = -15.31, *p* < 0.001), LIM-Cerebellum between-network connectivity (β = -1.97, *p* = 0.033), CON-Cerebellum between-network connectivity (β = 4.27, *p* = 0.008), Subcortical-Cerebellum between-network connectivity (β = 6.82, *p* = 0.003), and SMN node strength (β = 0.14, *p* = 0.001), with a cross-validated predictive accuracy of *R*^2^ = 0.26 (optimal λ = 0.63; RMSE = 1.21; SD = 0.36).

## 4 Discussion

The present study aimed to investigate financial abilities (FAs) in individuals with Parkinson’s disease and Mild Cognitive Impairment (PD-MCI), focusing on their cognitive and neurofunctional underpinnings. Overall, our findings indicate that, although basic and advanced FAs may share certain cognitive substrates, they also rely on distinct neurofunctional mechanisms in this population.

### 4.1 Cognitive correlates of FAs

In terms of cognitive correlates, basic FAs were significantly correlated with general cognition, particularly language abilities, while formal numerical competence emerged as a significant factor influencing basic FAs in the regression analysis. These associations are consistent with the notion that even simple financial transactions require integrating verbal comprehension and numerical reasoning. In particular, this finding extends previous studies reporting that patients with PD-MCI exhibit significant impairments in formal numerical competence (Burgio et al., 2022b), suggesting that these deficits may extend beyond abstract tasks and manifest in difficulties with routine financial activities, such as handling change or shopping for everyday goods. This underscores how subtle cognitive changes in individuals with PD-MCI can compromise autonomy in daily life.

In contrast, advanced FAs were linked to higher-order cognitive processes, including executive functions and social cognition. However, our modeling results further indicated that advanced financial tasks do not rely on a single cognitive domain but rather on the coordinated contribution of multiple cognitive mechanisms, encompassing language, memory, and formal numerical competencies. This pattern suggests that, as task complexity increases, patients may engage a broader range of cognitive strategies to sustain domain-specific abilities, potentially reflecting an adaptive but potentially less efficient processing approach. We might speculate that these patterns reflect compensatory mechanisms, which could explain the preserved performance in some advanced financial tasks despite underlying cognitive impairment.

Therefore, contrary to previous research that failed to identify significant associations between FAs and cognition in PD, either with or without MCI (Pirogovsky et al., 2013, 2014), our study revealed meaningful relationships between both basic and advanced FAs and specific cognitive domains. This discrepancy may reflect differences in patient characteristics and assessment tools, as our cohort may have been more cognitively compromised than those in earlier studies. Although direct comparisons between the cognitive profiles of the samples are difficult due to differences in the neuropsychological measures administered across studies, the patients with PD-MCI described in Pirogovsky’s study (Pirogovsky et al., 2014) were on average younger than those included in the present study (mean age of the PD-MCI subgroup: 69.2, SD: 7.1), with a shorter disease duration (mean years since diagnosis: 5.1, SD: 4.3), which may suggest overall a more preserved profile compared to our sample (Aarsland et al., 2021). Moreover, our use of the NADL-F, a measure specifically validated in the Italian context, could have offered a more ecologically valid and culturally sensitive assessment of everyday financial skills. Nevertheless, replication in other cultural contexts is needed to confirm the generalizability of these findings.

Interestingly, in models including only behavioral variables, both basic and advanced FAs were significantly related to demographic characteristics, such as participants’ educational levels. Education may serve as a proxy for an individual’s overall cognitive reserve, which is the ability to cope with neural damage through compensatory strategies (Nucci et al., 2012; Stern, 2009). In this view, our findings may suggest a potential role of cognitive reserve in maintaining everyday independence in individuals with PD-MCI, aligning also with prior studies on patients with MCI caused by other underlying conditions (Allegri et al., 2010; Poletti et al., 2011). Nevertheless, education was no longer a significant factor when neuroimaging variables were included in the models. This finding may reflect the fact that neuroimaging markers more directly capture disease-related neural changes, such as regional brain atrophy or alterations in functional networks, that have a stronger impact on cognitive performance than proxy measures of personal experiences, including education. Neuroimaging variables may therefore represent the downstream neural substrates through which education and cognitive reserve exert their effects, thereby attenuating the independent contribution of education once these brain-based measures are taken into account. Accordingly, future research should examine this topic more thoroughly by also considering other important factors, such as occupational complexity and participation in cognitively stimulating leisure activities, which can contribute to cognitive reserve.

### 4.2 Neuroimaging correlates of FAs

From a neuroimaging perspective, our results reveal distinct associations between financial abilities and large-scale brain network organization, supporting the notion that financial competence depends on a balance between network specialization, integration, and structural integrity.

Preserved performance in basic FAs was correlated with less integration within the DAN, as well as anti-correlation between the VIS and DAN, and between the SMN and DAN, suggesting a more efficient specialization of these networks. Such segregation is thought to support more efficient network specialization by limiting cross-network interference and allowing each system to operate more autonomously in accordance with its functional role (Bullmore and Sporns, 2009; Wig, 2017). Accordingly, the present findings may reflect a relatively preserved or compensatory network organization in which more autonomous and functionally distinct sensory and attentional processes support the efficient execution of perceptually guided financial tasks. These findings are consistent with previous evidence showing altered connectivity within and between large-scale brain networks in PD (Baggio et al., 2015; Putcha et al., 2015; Sang et al., 2015; Tessitore et al., 2019), specifically reporting reduced network segregation between attentional and sensory systems (Baggio et al., 2015; Olde Dubbelink et al., 2014). For instance, Baggio et al. (2015) reported widespread changes in resting-state network organization, particularly within visual and attentional circuits, which were associated with cognitive impairment in PD. Similarly, Putcha et al. (2015) demonstrated abnormal coupling among core neurocognitive networks, highlighting disrupted balance between network integration and segregation as a potential mechanism underlying cognitive deficits. In this context, our results extend these observations by suggesting that a more efficient functional specialization of sensory and attentional networks may help sustain basic financial skills, even in the presence of disease-related alterations.

Additionally, in regression models, basic FAs were associated with less integration within the somatomotor network, along with negative between-network connectivity between the SMN and subcortical regions. This result suggests adaptive segregation of motor loops from cognitive circuits, which may limit the spread of pathological motor-loop activity into executive and valuation networks. Moreover, this pattern aligns with prior evidence of altered sensorimotor-subcortical coupling in Parkinson’s disease (Sharman et al., 2013) and computational models linking basal ganglia dynamics to distributed network reconfiguration (Maith et al., 2021). Nevertheless, as these findings may reflect a confounding factor related to the motor symptomatology of PD (Tessitore et al., 2019; Tuovinen et al., 2018), future work should control for motor severity and dopaminergic state, and employ approaches (e.g., effective connectivity) able to determine whether this decoupling is causally compensatory or merely correlational with motor decline.

Regarding advanced FAs, we found associations with both structural preservation and functional mechanisms, with a prominent involvement of the cerebellum in the latter case. Concerning neuroanatomical metrics, the negative association between advanced FAs and CSF volume suggests that greater global brain atrophy is linked to poorer performance in more complex financial abilities. CSF volume is commonly considered an indirect marker of diffuse brain atrophy, reflecting cumulative neurodegenerative burden rather than focal gray matter loss (Ferrarini et al., 2008; Jack et al., 2000). Advanced financial abilities likely rely on the integration of multiple cognitive domains, as highlighted also by our exploratory correlational analysis, which are supported by distributed brain networks. As such, these higher-order abilities may be particularly sensitive to global structural deterioration, whereas more basic financial skills may remain relatively preserved until later stages of neurodegeneration. The absence of significant associations between voxel-based morphometry metrics and basic FAs, formal numeracy, or informal numeracy further supports the notion that these abilities are less dependent on focal gray matter integrity and may be sustained by more resilient or redundant neural systems. This pattern is consistent with evidence in Parkinson’s disease indicating that global measures of brain atrophy are more strongly related to complex cognitive outcomes than regional gray matter changes, particularly in non-demented or early stage patients (Aarsland et al., 2010; Mak et al., 2014).

Regarding neurofunctional mechanisms, preserved advanced FAs were linked to two complementary patterns of FC based on the cerebellum: (i) anti-correlation between the SVAN and cerebellar networks and between the LIM and cerebellar networks, and (ii) functional synchronization between frontoparietal-cerebellar and subcortical-cerebellar circuits. This dual configuration suggests that preserved financial decision-making relies on a finely balanced interplay between network specialization and cross-network communication. Opposing interactions of salience and limbic systems from the cerebellum may reflect a more modular and efficient organization, whereby the cerebellum operates with minimal interference from attentional and emotional systems. The involvement of limbic regions in financial processing has been reported in a previous study in patients with MCI related to AD pathology (Benavides-Varela et al., 2020), which suggested that altered limbic structures may lead to financial difficulties linked to emotional processing deficits. Our findings in patients with PD-MCI suggest that anti-correlation between limbic regions and the cerebellum may facilitate the execution of financial tasks, possibly by reducing emotional interference and enhancing goal-directed control. Similarly, given the present findings, we may speculate that stronger synchronization of cerebellar activity with frontoparietal and subcortical circuits likely might represent a compensatory mechanism supporting executive monitoring, working memory, and reward-based learning, which are essential for complex financial reasoning. These findings are consistent with evidence that the cerebellum forms reciprocal loops with both cortical control systems and basal ganglia structures, contributing to higher-order cognitive and affective processes in PD (Li et al., 2023; O’Callaghan et al., 2016). Similarly, our findings extend previous studies linking altered cerebellar FC with cortical and subcortical areas to the severity of non-motor symptoms (Pietracupa et al., 2024). In particular, our results highlight that preserved selective segregation and integration of cerebello-cortical and cerebello-subcortical networks support a high-level non-motor function, that is, financial competence. Collectively, these data support a model in which cerebellar network dynamics serve a key role in maintaining adaptive cognitive behavior despite dopaminergic and frontostriatal disruption. These results contribute to a growing body of evidence suggesting that changes in FC are not uniformly detrimental but may, in certain cases, represent adaptive network reorganization aimed at supporting everyday cognitive functioning in Parkinson’s disease.

Moreover, better advanced FAs were significantly associated with higher volumes of both GM and WM. This finding may suggest that structural integrity is fundamental to maintaining functional autonomy in daily tasks, and it also aligns with previous studies describing structural loss in PD patients with cognitive impairments (Aarsland et al., 2017).

Overall, based on our data, we speculate that basic FAs may be more sensitive to neurofunctional changes, while advanced FAs may be more linked to widespread functional alterations and structural damage. Indeed, prior studies reported that, in the earlier phases of neural alteration, functional reorganization and compensatory mechanisms may sustain adequate performance despite subtle connectivity disruptions (Putcha et al., 2016; Tessitore et al., 2019). However, as structural damage progresses (e.g., cortical thinning or atrophy), these compensatory processes may become insufficient, leading to deficits in complex, higher-order tasks, such as the financial ones. This interpretation aligns with longitudinal evidence indicating that functional changes often precede structural degeneration in PD, initially acting as adaptive responses to preserve cognitive performance (Sang et al., 2015). Nevertheless, since the present study acquired structural and functional neuroimaging data at a single time point, future studies should explore in greater depth the associations between financial competencies and longitudinal changes in neuroimaging metrics, also employing approaches that allow causal inferences.

Lastly, we observed that including structural and functional variables in the analyses improved model performance compared to models that used only cognitive measures. This emphasizes the importance of combining behavioral evaluations, such as neuropsychological assessments, with neuroimaging measurements to improve diagnosis and patient management.

### 4.3 Limitations and future directions

This study has some limitations that should be acknowledged. First, all data were collected at a single time point, making the findings correlational in nature. Consequently, we cannot draw conclusions regarding causal relationships or the progression of disease over time. Future studies should replicate and complement our findings by collecting longitudinal designs to better explore how cognitive and neural factors relate to the progression of financial difficulties in Parkinson’s disease. Second, the relatively small sample size and the availability of neuroimaging data for only a subset of participants limited the statistical power, preventing the use of more advanced analytical methods. Third, as the study was exploratory, no formal corrections for multiple comparisons were applied in order to limit the risk of type II errors, and potentially obscuring meaningful results. Nevertheless, this limitation should be considered when interpreting the results (Perneger, 1998; Rothman, 1990). Fourth, the use of a culturally specific measure of financial abilities (NADL-F) may limit the generalizability of our findings, which should be replicated across diverse cultural and linguistic contexts to verify their broader relevance. Moreover, the absence of more detailed clinical data, such as standardized assessments of motor impairment [e.g., the Unified Parkinson’s Disease Rating Scale (UPDRS)], limited our ability to fully explore the influence of motor symptoms on financial functioning. Motor symptoms may influence financial performance by affecting the execution of complex actions, processing speed, or attentional control, which are often required during financial tasks. Future studies should include specific measures to clarify the contribution of motor deficits in this crucial skill. Finally, the current study relied on resting-state and structural neuroimaging. Future research would benefit from task-based fMRI paradigms in which patients perform real-life financial tasks in the scanner, enabling a more direct investigation of the neural mechanisms supporting financial abilities in Parkinson’s disease.

## 5 Conclusion

In summary, the present study provides novel evidence on the cognitive and neurofunctional mechanisms that underlie financial abilities in individuals with Parkinson’s disease and Mild Cognitive Impairment.

Our findings show that basic financial tasks depend primarily on language and numerical competence and benefit from adaptive functional reorganization involving attentive, somatomotor and subcortical systems. In contrast, advanced financial competence engages distributed executive, mnemonic, and social-cognitive processes, supported by a cerebellar network configuration characterized by negative correlation with salience and limbic circuits and synchronization with frontoparietal and subcortical systems. This pattern suggests that effective financial decision-making depends on a dynamic balance between network specialization and compensatory integration.

Overall, these findings support a multidimensional model where both cognitive integrity and network organization jointly contribute to sustaining functional autonomy in PD-MCI. Assessing financial capacity through specific tools may therefore offer valuable insights into real-world functioning and serve as an early indicator of cognitive and neural decline. Future studies should replicate these results with larger samples and longitudinal designs, incorporating multimodal imaging approaches to clarify the temporal dynamics linking cognitive dysfunction, network reorganization, and everyday financial competence in Parkinson’s disease.

## Data Availability

The raw data supporting the conclusions of this article will be made available by the authors, without undue reservation.
