# Supplementary material for: Financial abilities in patients with Parkinson’s disease and mild cognitive impairment: unveiling cognitive and neurofunctional correlates of basic and advanced financial skills
Source: Front Aging Neurosci. 2026 Mar 4;18:1746491. doi: 10.3389/fnagi.2026.1746491 (PMC12995605; doi:10.3389/fnagi.2026.1746491)
Supplement: Supplementary file 1 [file Data_Sheet_1.docx]

Supplementary Material

# Supplementary Figures and Tables

**Table S1**. Type of pharmacological treatment administered to patients at the time of the study.

|  | N | % |
| --- | --- | --- |
| Levodopa | 33/33 | 100 |
| dopamine agonists | 16/33 | 48.5 |
| amantadine | 4/33 | 12.1 |
| anticholinergic agents | 0/33 | 0 |
| Adjunctive therapy with COMT inhibitors | 6/33 | 18.2 |
| Adjunctive therapy with MAO-B inhibitors | 16/33 | 48.5 |

**Table S2**. Demographic characteristics of the sample of healthy controls (N=242) used for z-scoring

|  | Range (min-max) | Mean | Standard deviation |
| --- | --- | --- | --- |
| Age, years | 35 - 94 | 65.10 | 13.93 |
| Education, years | 2 - 22 | 11.78 | 4.46 |
| Gender, N females* | -- | 136 | 56.20 |
| MMSE score | 24 - 30 | 28.48 | 1.53 |
| MoCA score | 17 - 30 | 25.78 | 2.86 |
| NADL-F total score | 9 - 22 | 17.79 | 2.84 |
| NADL-F basic FAs | 6 - 13 | 11.57 | 1.65 |
| NADL-F advanced FAs | 2 - 10 | 6.22 | 1.82 |

* N and percentages are reported. Acronyms: MMSE: Mini-Mental State Examination; MoCA: Montreal Cognitive Assessment; NADL-F: Numerical Activities of Daily Living - Financial; FAs: financial abilities

**Table S3**. Numerosities of healthy controls considered in the different subgroups stratified for age and education.

| Educational level | Age < 60 years | Age 60-69 years | Age 70-79 years | Age ≥ 80 years |
| --- | --- | --- | --- | --- |
| Lower education (≤8 years) | 19 | 20 | 38 | 11 |
| Higher education (>8 years) | 45 | 50 | 40 | 19 |

**Figure S1.** Spearman’s correlation coefficients between FAs components and functional neuroimaging metrics of within-network connectivity. Positive correlations are depicted in red, negative ones in blue. Significant correlations are highlighted with an asterisk (‘***’ p-value < 0.001; ‘**’ p-value < 0.01; ‘*’ p-value < 0.05). Acronyms: WM: white matter; GM: grey matter; CSF: cerebrospinal fluid; TIV: total intracranial volume.


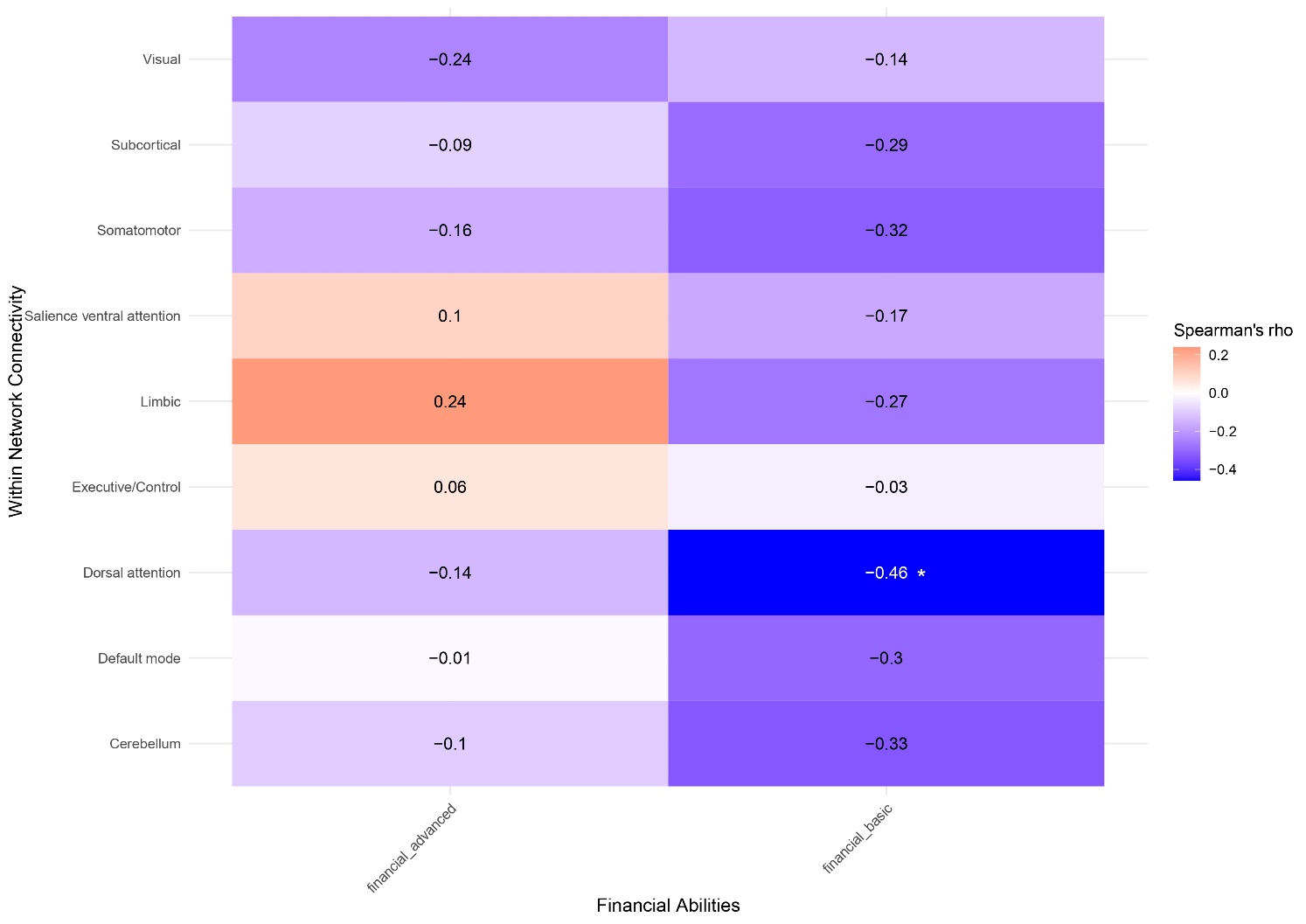


**Figure S2.** Spearman’s correlation coefficients between FAs components and functional neuroimaging metrics of between-network connectivity. Positive correlations are depicted in red, negative ones in blue. Significant correlations are highlighted with an asterisk (‘***’ p-value < 0.001; ‘**’ p-value < 0.01; ‘*’ p-value < 0.05). Acronyms: SMN: somatomotor network; VIS: visual network; DAN: dorsal attention network; SVAN: salience ventral attention network; LIM: limbic network; CON: Control network; DMN: default mode network; bn-FC: between-network functional connectivity.

**
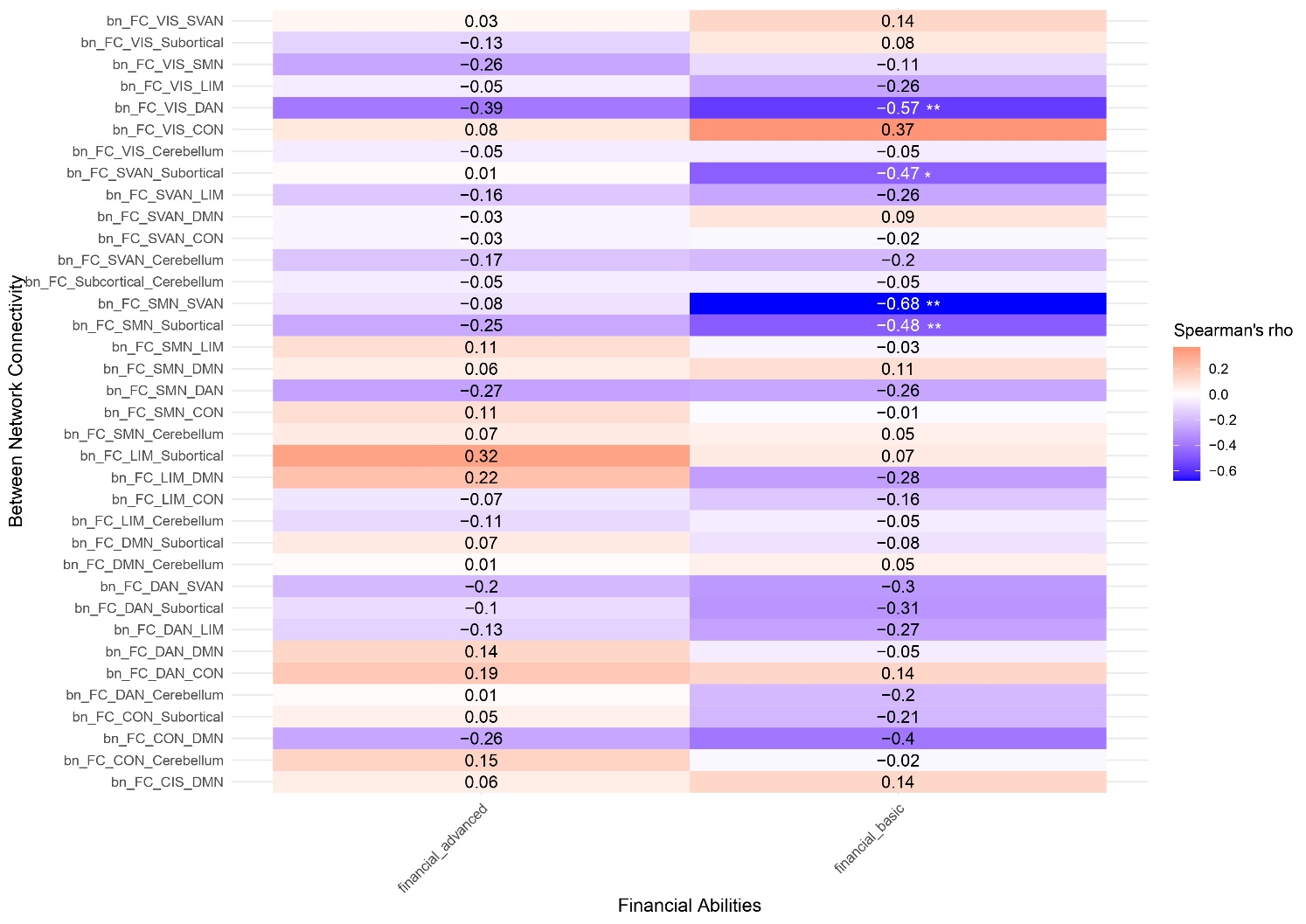
**
